# Exploring variation in quality of care and clinical outcomes between neonatal units: a novel use for the UK National Neonatal Audit Programme (NNAP)

**DOI:** 10.1136/bmjoq-2022-002017

**Published:** 2022-10-17

**Authors:** Abdul-Qader Tahir Ismail, Elaine M Boyle, Sam Oddie, Thillagavathie Pillay

**Affiliations:** 1 Department of Health Sciences, University of Leicester, Leicester, UK; 2 Bradford Institute for Health Research, Bradford Teaching Hospitals NHS Foundation Trust, Bradford, UK

**Keywords:** Healthcare quality improvement, Quality measurement, Quality improvement methodologies

## Abstract

Neonatology is a relatively new specialty, in which much of the practice remains non-evidence based. Variation in the quality of care delivered is recognised but measuring this is challenging. One possible indicator of this is variation in practice. For more than a decade, the National Neonatal Audit Project (NNAP) has described variation in practice between UK neonatal units in relation to its annually reviewed audit measures. These are based on evidence based national standards or developed by a consensus method and have become de facto measures defining good quality of neonatal healthcare within the UK. In this article we explore the practicality of using the NNAP to look for associations between quality of care and outcomes. This would not be to validate the measures but could help towards a better understanding of the reasons underlying recognised variation in outcomes, even between neonatal units of the same designation.

## Introduction: measuring quality of care

As healthcare providers and consumers, we all desire good quality of care but defining what this means is not straightforward. As physicians, we commonly believe good quality of care means achieving better outcomes by practising evidence-based medicine while delivering optimal patient experience. Processes of care supported with strong evidence (eg, from randomised controlled trials and systematic reviews) are often incorporated into clinical guidelines and standards. Adherence to such guidance (monitored and reinforced through audit cycles) is assumed to represent delivery of ‘good quality of care’, for example, administration of first dose of antibiotics within 60 min to patients with sepsis,^1^ or administration of antenatal corticosteroids to pregnant women who are expected to deliver a preterm baby.^2^ However, many areas of medicine have a limited evidence base and variation in clinical practice is common, especially in relatively new specialties such as neonatology.^3 4^ Variation in practice may imply variation in the quality of care we deliver. One way of measuring quality of care is to decide on best practice (based on available evidence) and, within that context, describe variation in practice that exists. Depending on the strength of the evidence supporting ‘best practice’ we might also expect to find an association between ‘quality of care’ and outcomes.

In this review, we consider how data reflecting variation in neonatal practice analysed by the UK National Neonatal Audit Programme (NNAP) could be used as a surrogate for measuring quality of neonatal healthcare and analysing its relationship with clinical outcomes.

## Analysing variation in neonatal practice: the UK NNAP

The NNAP commenced in 2007 to ‘*help neonatal units improve care for babies and their families by identifying areas for quality improvement in relation to the delivery and outcomes of care’.*^5^ NNAP is funded by the Department of Health and administered by the Healthcare Quality Improvement Partnership. The tender to deliver the programme was awarded to the Royal College of Paediatrics and Child Health. Opportunities for quality improvement are considered by examining adherence to annually reviewed standards or audit measures based on published national standards or developed by a consensus method. Table 1 shows the 2022 audit measures.^6^ ‘Adherence’ to an audit measure has previously been determined using data from the National Neonatal Research Database. Data are now obtained directly from an electronic patient record supplier. The publicly available annual NNAP report provides comparison charts for each unit’s adherence with each measure.

**Table 1:** 2022 NNAP audit measures^6^

Optimal perinatal care	**Antenatal steroids** Does a mother who delivers a baby between 23 and 33 weeks’ gestational age receive a full course of antenatal corticosteroids within 1 week prior to delivery?
	**Antenatal magnesium sulfate** Does a mother who delivers a baby below 30 weeks’ gestational age receive magnesium sulfate in the 24 hours prior to delivery?
	**Birth in a centre with a neonatal intensive care unit (NICU)** Is a baby: Born at less than 27 weeks’ gestational age? Or less than 800 g at birth? Or born as a multiple at less than 28 weeks’ gestational age delivered in a maternity service on the same site as a designated NICU?
	**Deferred cord clamping for very preterm babies** Does a baby born at less than 34 weeks’ gestational age have their cord clamped at or after 1 min?
	**Promoting normal temperature on admission for very preterm infants** Does a baby born at less than 32 weeks’ gestational age have a first temperature on admission which is both between 36.5°C and 37.5°C and measured within 1 hour of birth?
	**Type and duration of respiratory support** What proportion of babies born at less than 32 weeks’ gestation only receive non-invasive respiratory support during the first week of life?
Parent partnership	**Parental consultation within 24 hours of every admission** Is there a documented consultation with parents by a senior member of the neonatal team within 24 hours of admission?
	**Parental presence at consultant ward rounds** For a baby admitted for more than 24 hours, was at least one parent included a consultant ward round? What proportion of consultant-led ward rounds had at least one parent included?
Care processes	**Breastmilk feeding at 48 hours** Does a baby born at less than 34 weeks’ gestational age receive any of their own mother’s milk in the first 2 days of life?
	**Breastmilk feeding at day 14** Does a baby born at less than 34 weeks’ gestational age receive any of their own mother’s milk at day 14 of life?
	**Breastmilk feeding at discharge to home** Does a baby born at less than 34 weeks’ gestational age receive any of their own mother’s milk at discharge to home from a neonatal unit?
	**On-time screening for retinopathy of prematurity** Does a baby born at less than 31 weeks’ gestational age or weighing less than 1501 g at birth undergo the first ROP screening according to the guideline?
	**Follow-up at 2 years of age** Does a baby born at less than 30 weeks’ gestational age receive medical follow-up at 2 years’ gestationally corrected age (18–30 months’ gestationally corrected acceptable age range)? Does a baby have complete results of a structured assessment recorded?
	**Nurse staffing on neonatal units** What proportion of nursing shifts are numerically staffed according to guidelines and service specification?
Clinical outcomes	**Bloodstream infection** Does an admitted baby have one or more episodes of bloodstream infection, characterised by one or more positive blood cultures taken, after 72 hours of age?
	**Bronchopulmonary dysplasia** Does an admitted baby born at less than 32 weeks’ gestational age develop bronchopulmonary dysplasia (BPD) or die?
	**Necrotising enterocolitis** Does an admitted baby born at less than 32 weeks’ gestational age meet the NNAP surveillance definition for necrotising enterocolitis (NEC) on one or more occasions?
	**Neonatal preterm brain injury** Does a baby born at less than 32 weeks’ gestational age experience any of the following forms of brain injury? Germinal matrix/intraventricular haemorrhage. Posthaemorrhagic ventricular dilatation. Cystic periventricular leukomalacia.
	**Mortality to discharge in very preterm babies** Does a baby born between 24 and 31 weeks’ gestational age inclusive die before discharge to home, or 44 weeks’ postmenstrual age (whichever occurs sooner)?

NNAP, National Neonatal Audit Programme; ROP, retinopathy of prematurity.

Some of the audit measures have set standards (eg, 90% for promoting normal temperature on admission of very preterm babies, 85% for birth of babies born <27 weeks of gestation in a centre with a neonatal intensive care unit (NICU)), while others do not and are described as ‘benchmarking’ (eg, delayed cord clamping, parental presence on consultant ward rounds). For the former, part of the purpose of the NNAP is in identifying outliers. Units have an opportunity to correct their data, which is represented to them at the end of the data year. Using that final version of the data, outlier units are identified, and if these are ‘alarm’ outliers (ie, 3 SDs below the set standard) then a process of outlier management is instigated. This involves the local production of an action plan, and the relaying of outlier status to various parties including the Care Quality Commission (CQC, or equivalent in Scotland and Wales). The CQC will expect to see action plans of how they aim to address their outlier status; adherence to which will help avoid regulatory action.

Therefore, for over a decade, NNAP audit measures have described variation in clinical practice across UK neonatal units in relation to the audit measures, which have become *de facto* standards defining good quality of neonatal healthcare.

## Why investigate associations between quality of care and clinical outcomes?

Associations between provision of good quality of care and outcomes might be expected. However, since the NNAP audit measures were introduced, these data have never been used to examine associations between adherence with the measures and mortality and major morbidity outcomes. Surely this would be the gold standard for assessing whether the measures are valid (ie, truly measure quality of care).

To challenge this assumption, consider the scenario where there is strong evidence supporting a practice that is used as a quality-of-care measure (eg, antenatal administration of magnesium sulfate to pregnant women who are expected to deliver a preterm baby^7^). Does it matter whether we can find a correlation between adherence of healthcare providers and outcomes for their patients? If we do, it supports the evidence originally used as the basis for choosing it as a quality-of-care measure. If there is no such correlation, does this therefore suggest the practice does not constitute good quality of care and should not be the basis of a guideline or standard? Alternatively, would we have to assume that the analysis might be affected by unmeasured confounding?

This is more challenging considering quality-of-care measures relating to aspects of care that have less robust evidence linking to clinical outcomes (eg, correct timing of retinopathy of prematurity screening, or parental presence on consultant ward rounds). In such cases, where an association between adherence and improved outcomes is plausible but has not been proven and cannot easily be demonstrated, then this could be interpreted in different ways, one of which might be to dismiss it as not being a valid measure of care quality.

Therefore, even though we might expect healthcare providers that strive to deliver good quality of care to have better outcomes for their patients, this may not always be the case. It is when quality-of-care measures are chosen that the analysis and decision needs to be made about whether they are valid. They cannot be validated through seeking associations with outcomes; so why investigate associations between quality of care and outcomes?

## Variation in practice can help explain variation in clinical outcomes between neonatal units

It is well documented that, even when comparing neonatal units of the same level/designation, outcomes can vary on a national and even regional level.^8–12^ The MBRRACE-UK collaboration (Mothers and Babies: Reducing Risk through Audits and Confidential Enquiries across the UK)^13^ analyses data on all UK births and perinatal/neonatal deaths. Crude hospital-based mortality rates are ‘stabilised’ and ‘adjusted’. Stabilisation involves allowing for fluctuations in mortality rates due to chance, which are more pronounced in smaller hospitals with lower number of deliveries. Adjustment considers maternal and neonatal risk factors that can result in increased mortality rates in areas of social deprivation, and in large hospitals that serve as referral units for high-risk pregnancies. Deaths due to congenital anomalies are also excluded. The 2021 report shows that when comparing 2019 neonatal mortality rates for NICU with surgical provision, even after adjustment and stabilisation, they varied from 1.58 to 4.49/1000 live births. Similar variation was seen for other types of units, categorised by designation or volume of patients.

Therefore, exploration of variation in the quality of care delivered by neonatal units may help explain the differences in clinical outcomes that remain even after statistical methods such as those described above. In the subsequent sections we will discuss some of the general considerations relevant to conducting such an analysis.

## Classifying NNAP audit measures according to structure, process and outcomes of healthcare

**Figure 1:**
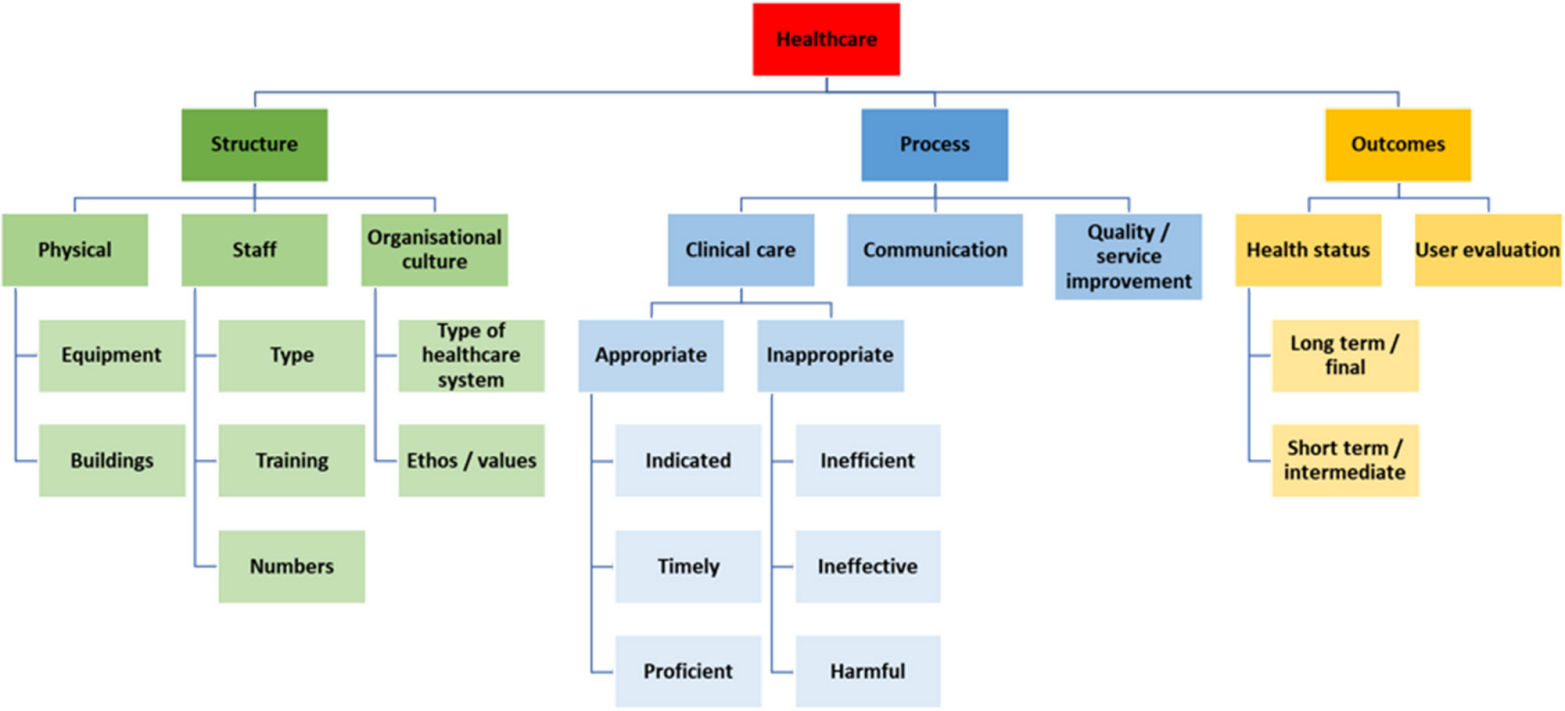
Aspects of healthcare classified according to structure, process and outcomes.

It is helpful to classify the NNAP audit measures according to the widely used framework introduced by Donabedian in the 1960s of the three healthcare domains: structure, process and outcomes (figure 1, table 2).^14^ Some of the measures may arguably straddle two, or even all these domains, and this is especially true for those relating to the evolving dynamic of parental partnership in care.

**Table 2:** Categorisation of 2022 NNAP audit measures by whether they primarily relate to structure, processes or outcomes of healthcare

Structure	Process	Outcome	
		Intermediate	Final
Minimising inappropriate separation for term and late preterm babies. Two-year follow-up. Parental presence at consultant ward rounds. Nurse staffing. Birth in centre with NICU.	Antenatal steroids. Antenatal magnesium sulfate. Parental consultation within 24 hours of admission. Breastmilk feeding at 48 hours. Deferred cord clamping. Type and duration of respiratory support.	Promoting normal temperature on admission. Bloodstream infection. On-time screening for ROP. Breastmilk feeding at day 14. Breastmilk feeding at discharge to home. Neonatal preterm brain injury.	NEC. BPD. Mortality.

BPD, bronchopulmonary dysplasia; NEC, necrotising enterocolitis; NICU, neonatal intensive care unit; NNAP, National Neonatal Audit Programme; ROP, retinopathy of prematurity.

It is tempting, when considering quality-of-care measures, to choose long-term or final clinical outcomes. However, identification of suboptimal outcomes or a difference between providers does not indicate how structure or processes of the healthcare system should change to ameliorate this. In part, this is because these outcomes can occur many years after first contact with healthcare, as opposed to short-term or intermediate outcomes, which can be useful to evaluate care quality and disease progression in the intervening time period. However, even for short-term outcomes, factors other than care provided can have an effect. These include age, sex, socioeconomic and health status, illness severity, treatment compliance, etc. While adjusted analyses are useful for such known confounders, unknown confounders can only be properly compensated for in a randomised trial, which is not always feasible or ethical. Therefore, using outcomes (especially long-term or final outcomes) as quality-of-care measures can lead to invalid comparisons, inaccurate conclusions and vague or unimplementable recommendations.

Healthcare structure and processes of care may therefore be more suitable for use as quality-of-care measures. This allows us to define what we believe constitutes good quality healthcare, with the primary determinant often being the strength of supporting evidence linking to outcomes. However, for many aspects of healthcare, it is not possible to find robust or direct evidence linking to outcomes. This may be due to rarity of a condition and inability to conduct large-scale, adequately powered studies or loss of equipoise due to previous observational data and personal or anecdotal experience. Other aspects of healthcare relate to structural systems necessary to allow delivery of good quality care (eg, timing of appointments and screening tests). To identify and use such measures, lower levels of evidence can be relied on (ie, observational studies down to consensus of expert opinion).

## Using adherence with and data completion for the NNAP audit measures as quality-of-care measures

There are two ways we can analyse the NNAP audit measures’ data for this purpose: adherence and data completion.

It seems intuitive that there should be some link between the audit measures used as quality-of-care measures and clinical outcomes of interest. For example, if interested in cerebral palsy and neurodevelopmental outcomes, we might look for correlations with units meeting the standards for magnesium sulfate administration. However, we must consider that NNAP audit measures have formed national guidance on what constitutes good neonatal care for over a decade. Therefore, units complying with the audit measures may have overall better outcomes, not because of the specific measures for which they are meeting standards but because this reflects an organisational culture of striving for ongoing quality improvement, as measured by their efforts to meet national standards. This involves both adherence with the audit measures in terms of fulfilling their requirements, and sufficient data completion to allow accurate monitoring of that adherence. Therefore, rather than an analysis of individual measures and related outcomes, it may be appropriate to conduct an analysis on whether those units with better overall adherence or data completion have better clinical outcomes.

This approach also requires us to consider that the standards or benchmarks set within individual audit measures for a specific gestational age group can have wider implications for overall quality of care delivered by units. Otherwise, for example, for the audit measure relating to antenatal steroids we would only look for associations with outcomes for babies born between 23 and 33 weeks of gestation. This would make it difficult to look at adherence or data completion for a combination of audit measures, since several apply only to babies of different gestational age ranges. In other words, analysis would be needed at a unit level (regarding adherence/data completion and outcomes) rather than at a patient level.

Using data collected over several years would increase the sensitivity of analyses, especially if interrogating outcomes by each gestational week of birth. This can reduce sample sizes due to low incidence of clinically significant outcomes and smaller numbers of babies born at lower gestational ages. However, NNAP audit measures are annually reviewed, and changes made regarding definitions and introduction of new measures (eg, the temperature audit measure was changed from applying to babies born <29 weeks of gestation in 2014 to <32 weeks from 2015 onwards, and the audit measure relating to nurse staffing was introduced in 2018). Furthermore, the healthcare environment of a neonatal unit can vary from year to year with focus on different areas for quality improvement, for example, following the introduction of the Commissioning for Quality and Innovation framework aimed at reducing inappropriate admission of term babies to neonatal units.^15^ These opposing factors would need consideration when deciding on the duration of a study. A possible compromise would be to use audit measures that have remained constant over several years.

## Using non-clinical outcomes as quality-of-care measures

The subjective experience of those receiving and delivering care, that is, parents and healthcare staff, forms important non-clinical outcomes that could be used as surrogates for quality of care to provide a more holistic picture. We have not explored this here since the NNAP does not collect this type of data, nor is there currently a suitable national alternative. If such data were available, it would be interesting to look for associations between unit parent/staff scores, adherence/data completion for NNAP audit measures and outcomes.

## Conclusion

In the UK, the NNAP is a well-established system for measuring and comparing quality of neonatal care. However, it has never been used to examine associations between adherence with and data completion for the measures and mortality and major morbidity outcomes. The purpose of doing such an analysis would be to see if it can help explain the variation in clinical outcomes that is known to exist between neonatal units, even of the same designation. In this review, we have explored how to approach such an analysis. This approach might usefully complement existing quality improvement methodologies and tools to understand root causes. In this article, we have focused on neonatal healthcare; however, this concept could be extended to any specialty in which a robust source of national audit data is available that describes variation in practice and could be used to look for associations with outcomes.
